# Perirenal Fat Volume Is Positively Associated With Serum Uric Acid Levels in Chinese Adults

**DOI:** 10.3389/fendo.2022.865009

**Published:** 2022-05-06

**Authors:** Ming Jiang, Menghuan Li, Cuiying Liu, Lei Jing, Qiong Huang, Tingting Wu, Xiangqing Kong, Jing Liu

**Affiliations:** ^1^Department of Cardiology, The First Affiliated Hospital of Nanjing Medical University, Nanjing, China; ^2^Department of Cardiology, The Medical School of Southeast University, Nanjing, China; ^3^Department of Ultrasound, The First Affiliated Hospital of Nanjing Medical University, Nanjing, China

**Keywords:** serum uric acid, perirenal fat, visceral fat, ultrasonography, adipose tissue, inflammation

## Abstract

**Background:**

Visceral fat has been considered an important risk factor of elevated serum uric acid (SUA). Perirenal fat is a unique visceral fat around the kidneys that has special morphological and physiological features while its relationship with SUA remains incompletely elucidated. This study aimed to assess the association between perirenal fat volume (PrFV) and SUA.

**Methods:**

A cross-sectional study was conducted in 102 subjects aged ≥ 18 years old recruited from Nanjing,China. The clinical characteristics including age, sex, drinking behavior, history of hypertension, body mass index, waist circumference, total cholesterol, fast plasma glucose, urea, serum creatinine, C-reactive protein, and SUA were recorded. PrFV was measured by ultrasonography. Multivariate linear models and the restricted cubic spline were used to investigate the association between PrFV and SUA.

**Results:**

The median age of this study population was 52.5 (42.0-60.0) years and 56.9% were female. The median value of SUA was 5.73 mg/dL (4.58-6.80 mg/dL). The subjects were divided by PrFV tertiles and we found that the subjects in the highest PrFV tertile had a higher level of SUA compared to those in the lowest tertile (β=1.86, 95%CI 1.23–2.48, *P* for trend <0.001).The positive association also remained after adjustment for potential covariates (tertile3 versus tertile1: β=0.99, 95%CI 0.35-1.63, *P* for trend =0.005). There was an increase of approximately 0.53 mg/dL in SUA per 1-fold increase in PrFV (β=0.53, 95%CI 0.02-1.04, *P* for nonlinearity = 0.637).

**Conclusion:**

Our results confirmed a positive independent relationship between PrFV and SUA in Chinese adults. This study suggested that perirenal fat might constitute a potential risk factor for elevated serum uric acid levels.

## Introduction

Hyperuricemia is a metabolic disease characterized by elevated serum uric acid (SUA), the end-product derived from purine metabolism (1). With changes in lifestyle and diet in modern society, the increasing prevalence and incidence of hyperuricemia are likely to contribute substantially to the increasing burden of public health worldwide (2). Furthermore, it has already been associated with many diseases, including hypertension (3), cardiovascular diseases (4), diabetes mellitus (5, 6), metabolic syndrome (7), non-alcoholic fatty liver disease (8), and chronic kidney disease (9). Knowing risk factors for hyperuricemia is of critical importance to allow early interventions and mitigate cardiovascular disease burden.

Visceral adipose tissue has been regarded as an endocrine organ involved in numerous physiological processes such as lipid and glucose metabolism (10, 11). Recently, increasing evidence has suggested that visceral fat accumulation is correlated with elevated SUA levels. Takahashi et al. reported that the visceral fat area independently contributed to an elevated level of SUA in healthy men and was more accumulated in patients with gout (12). Zong and colleagues found that visceral fat was associated with SUA levels in people with type 2 diabetes mellitus (13). Yamada’s results were consistent with previous studies and demonstrated that the association between visceral fat and SUA was independent of lifestyle factors, including physical activity level, alcohol consumption, and smoking (14). Furthermore, visceral fat was more closely associated with high SUA levels compared to subcutaneous fat (15). Reduced visceral fat area was associated with a decrease in SUA level (16). Based on such evidence, visceral fat is suggested to be a significant risk marker for SUA.

Perirenal fat is a special visceral fat that is stored around the kidneys and has active abilities in metabolism regulation and adipokine secretion. The fibrous membrane and renal fascia separate perirenal fat from the other neighboring adipose tissues including pararenal fat and renal sinus fat (17). Due to its unique anatomy and physiological features, perirenal fat is known to be associated with many metabolic indexes (18–20). However, the association between perirenal fat and SUA has not been fully evaluated. In clinical studies, ultrasonography and computed tomography (CT) are widely used to measure the amount of perirenal fat. Compared to CT, ultrasonography is more convenient, easy-to-perform and without ionizing radiation. However, previous studies only measured a single thickness of perirenal fat on a specific layer *via* ultrasonography, which was insufficient to quantify the perirenal fat content (21, 22).

The inferior perirenal fat has the most abundant fat considering the non-uniform distribution of the perirenal fat (17). In our study, the inferior perirenal fat was measured longitudinally, transversally, and anterior-posteriorly by ultrasound, which was a more robust and precise approach to quantifying the amount of perirenal fat. Then, we conducted a cross-sectional study to explore the association between PrFV and SUA among Chinese adults.

## Methods

### Study Population

The study population consisted of 84 subjects from a randomized control trial on a novel cholesterol-lowering therapy of perirenal fat modification mediated by a noninvasive high-intensity focused ultrasound (HIFU) registered at ClinicalTrials.gov (NCT04223557) and 18 subjects enrolled in a pilot study that evaluated the safety of noninvasive HIFU-mediated perirenal fat modification for the treatment of hypertension. All adult subjects (over 18 years old) were consecutively recruited from May 2020 to June 2021 at the First Affiliated Hospital of Nanjing Medical University, Nanjing, China.

All subjects were interviewed for demographic information and medical history. Patients with diabetes mellitus, chronic kidney disease [defined as an estimated glomerular filtration rate (eGFR) < 60 mL/min/1.73 m^2^], renal morphological abnormalities, history of kidney surgeries, antihyperuricemic medication, antihyperlipidemic medication, diuretics, cancer, and present pregnancy were excluded from the study. The study protocols were approved by the Institutional Review Board of the First Affiliated Hospital of Nanjing Medical University and were performed following the Declaration of Helsinki. A written Informed consent was obtained from each subject.

### Anthropometric Evaluation

All subjects were weighed in light clothing without shoes. Body height and weight were measured by KF-1328LED, a human height-weight scale (Kaifeng Group Co., Zhejiang, China), and body mass index (BMI) was calculated as kilograms per square meter. Waist circumstance (WC) was measured at the midpoint between the lower border of the rib cage and the top of the lateral border of the iliac crest.

### Laboratory Measurements

Blood samples were collected after an overnight fast of at least 8 hours. Total cholesterol (TC), triglycerides (TG), fast plasma glucose (FPG), urea, serum creatinine (SCr), and SUA were measured by standard enzymatic methods using an automatic biochemistry analyzer (Au5800, Beckman Coulter, United States). C-reactive protein (CRP)was measured by a nephelometric method using an automatic immunochemistry analyzer (Immage 800, Beckman Coulter, United States). eGFR was calculated using the MDRD formula (23): eGFR(ml/min/1.73m^2^) =186×(SCr/88.402) ^−1.154^ ×age^−0.203^ × (0.742 if female).

### Ultrasound Assessment

**Figure 1:**
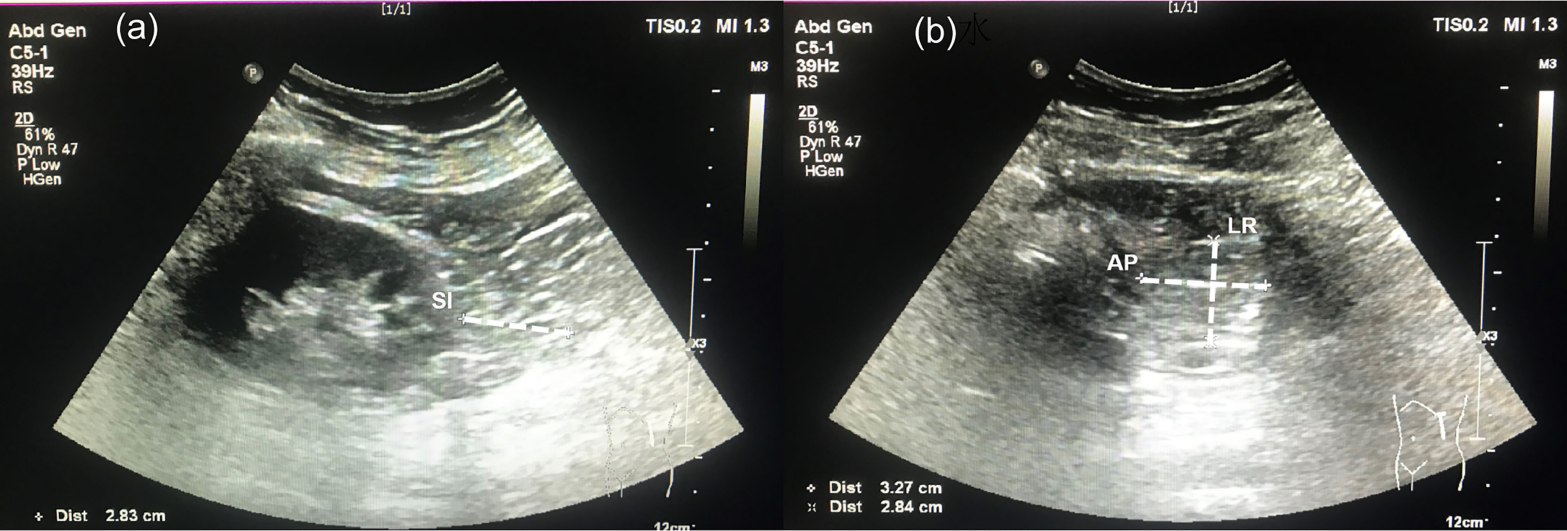
Ultrasonographic measurement for perirenal fat volume. **(A)** A long-view of the inferior perirenal fat. Superior-inferior (SI) diameter was defined as the maximal thickness between the fibrous membrane to the renal fascia. **(B)** A short-view of the inferior perirenal fat. The left-right (LR) diameter and anterior-posterior (AP) diameter were measured through the maximal short-axis view of the inferior perirenal fat.

A single skilled operator who was blind to subject information performed the assessment using a Philips EPIQ7 ultrasound system (Philips Ultrasound, Bothell, WA, USA). The subjects underwent an examination in a lateral decubitus position. At the inferior pole of the kidney through the long-axis section, the maximal thickness between the fibrous membrane to renal fascia was defined as the superior-inferior (SI) diameter. Meanwhile, the maximum left-right diameter (LR) and anterior-posterior diameter (AP) were measured through the short-axis view of the inferior perirenal fat. PrFV was calculated using SI, LR, and AP diameters. After obtaining bilateral PrFV values, the average value was used for analysis. The intraoperator coefficient of variation obtained by 2 separate PrFV measurements in 10 subjects was 3.3%. An ultrasound image of three diameters is shown in **Figure 1**.

### Statistical Analysis

The study population was divided into three groups based on tertiles of PrFV (Tertile1 ≤ 58.18cm^3^, 58.18<Tertile2 ≤ 100.04cm^3^, Tertile3>100.04cm^3^). Continuous variables with a skewed distribution were expressed as a median and interquartile range. The nonparametric Kruskal-Wallis test for skewed variables and the chi-square test for categorical variables were used to evaluate differences between groups. Spearman’s *r* test was calculated to assess the simple correlations between SUA and other clinical variables. Multivariate generalized linear models were performed to assess the associations between the tertiles of PrFV and SUA— Model 1, unadjusted; Model 2, adjusted for age, sex; and Model 3, adjusted for age, sex, WC, SCr, CRP (covariates were selected according to the influence to β value of PrFV and the Akaike information criterion). Additionally, we performed a sensitivity analysis to assess whether TC, TG, and BMI values influenced the association between PrFV and SUA. The linear trends tests were performed by examining each PrFV tertile as an ordinal variable. Then, we used a generalized linear model and restricted cubic spline with 3 knots to explore the association between PrFV (per 1-fold increase) and SUA with the same covariates. Nonlinearity (*P* for nonlinearity) was tested by Wald X^2^ tests. Statistical analyzes were performed using the IBM SPSS Statistics software package version 23 and the R software version 3.6.3. Statistical significance was defined as *P* ≤ 0.05.

## Results

### Clinical Characteristics of the Study Population

**Table 1** shows the characteristics of the overall study subjects and stratified by tertiles of PrFV. The study population included 102 Chinese adults with 56.9% female and a median age of 52.5 (42.0-60.0) years. The median value of SUA was 5.73 mg/dL (4.58-6.80 mg/dL). Compared to the other two groups, subjects in the lowest PrFV tertile were more likely to be female (*P*<0.05). There were significant differences in BMI, WC, TG, SCr, CRP, and SUA among the three groups. Subjects with higher PrFV had higher BMI, WC, and SUA compared to those with lower PrFV(*P*<0.001). And subjects in the highest PrFV tertile had higher levels of TG and CRP than the other two groups (*P*<0.05). Moreover, subjects in the lowest PrFV group had the lowest SCr compared to those in the other two groups (*P*<0.01 and *P*<0.05, respectively). However, no significant differences were found in age, current drinker, hypertension, TC, FPG, Urea, or eGFR among the three groups.

**Table 1:** Characteristics of the study population stratified across PrFV tertiles.

	Total (n=102)	Tertile1 (n=34)	Tertile2 (n=34)	Tertile3 (n=34)	*P* ^a^
Age, year	52.5 (42.0-60.0)	56.5 (45.3-60.8)	52.5 (42.5-60.0)	47.0 (40.75-57.0)	0.107
Female%	58 (56.9)	29 (85.3)	18 (52.9) *	11 (32.4) *	**<0.001**
Current drinker%	29 (28.4)	7 (20.6)	9 (26.5)	13 (38.2)	0.259
Hypertension%	49 (48.0)	13 (38.2)	19 (55.9)	17 (50.0)	0.333
BMI, kg/m^2^	25.3 (23.8-27.6)	24.1 (21.6-25.1)	25.9 (3.7-26.7) *	27.7 (25.1-29.8) ***^†^	**<0.001**
WC, cm	88.5 (81.9-94.3)	82.0 (76.0-87.0)	87.0 (81.1-92.3)	95.5 (89.7-104.0) ***^†††^	**<0.001**
TC, mmol/L	5.97 (5.55-6.53)	6.15 (5.61-6.79)	5.85 (5.53-6.32)	5.96 (5.50-6.60)	0.116
TG, mmol/L	1.52 (1.11-2.37)	1.29 (0.84-1.89)	1.58 (1.21-2.20)	1.99 (1.28-2.84) **	**0.010**
FPG, mmol/L	5.19 (4.87-5.78)	5.20 (4.87-5.81)	5.03 (4.87-5.53)	5.35 (4.82-5.80)	0.463
Urea, mmol/L	4.99 (4.47-5.83)	4.73 (4.15-5.77)	4.87 (4.34-6.07)	5.32 (4.78-5.94)	0.127
SCr, μmmol/L	66.10 (55.48-75.20)	58.20 (54.18-67.05)	71.10 (61.00-76.18) **	68.00 (57.20-83.73) *	**0.003**
eGFR, mL/min*1.73 m^2^	104.24 (89.21-116.55)	107.44 (91.44-116.06)	96.06 (85.65-115.88)	112.69 (86.97-125.06)	0.221
CRP, mg/L	1.81 (1.35-2.84)	1.54 (1.15-2.20)	1.60 (1.19-2.30)	2.35 (1.65-4.89) *^†^	**0.007**
SUA, mg/dL	5.73 (4.58-6.80)	4.71 (4.26-5.80)	5.65 (4.22-6.59)	6.76 (5.83-7.56) ***^††^	**<0.001**

Values are expressed as median (interquartile range) or number (%).

PrFV, perirenal fat volume; BMI, body mass index; WC, waist circumference; TC, total cholesterol; TG, triglycerides; FPG, fast plasma glucose; SCr, serum creatinine; eGFR, estimated glomerular filtration rate; CRP, C-reactive protein; SUA, serum uric acid.

a Using the Kruskal-Wallis test for continuous variables, the chi-square test for categorical variables.

***P < 0.001, **P < 0.01, *P < 0.05 vs Tertile1, ^†††^P < 0.001, ^††^P < 0.01, ^†^P < 0.05 vs Tertile2.

Statistically significant p-values were expressed with bold.

### Correlations of SUA and Clinical Variables

Then we analyze the simple correlations of SUA and clinical variables including age, BMI, WC, TC, TG, FBG, Urea, SCr, eGFR, CRP and PrFV (**Table 2**). SUA was strongly and negatively associated with age (*r*=-0.288, *P*=0.003) and TC (*r*=-0.217, *P*=0.029). And SUA was strongly and positively associated with BMI (*r*=0.419, *P*<0.001), WC (*r*=0.562, *P*<0.001), TG (*r*=0.440, *P*<0.001), SCr (*r*=0.489, *P*<0.001), CRP (*r*=0.222, *P*=0.029) and PrFV (r=0.517, *P*<0.001), whereas no correlation was found between SUA and FBG (*r*=0.085, *P*=0.398), Urea (*r*=0.122, *P*=0.222) and eGFR (*r*=-0.135, *P*=0.177).

**Table 2:** Correlations between SUA and clinical variables in the study population.

Variables	SUA, mg/dL	
	*r*	*P*
Age, year	-0.288	**0.003**
BMI, kg/m^2^	0.419	**<0.001**
WC, cm	0.562	**<0.001**
TC, mmol/L	-0.217	**0.029**
TG, mmol/L	0.440	**<0.001**
FPG, mmol/L	0.085	0.398
Urea, mmol/L	0.122	0.222
SCr, μmmol/L	0.489	**<0.001**
eGFR, mL/min*1.73 m^2^	-0.135	0.177
CRP, mg/L	0.222	**0.029**
PrFV, cm^3^	0.517	**<0.001**

BMI, body mass index; WC, waist circumference; TC, total cholesterol; TG, triglycerides; FPG, fast plasma glucose; SCr, serum creatinine; eGFR, estimated glomerular filtration rate; CRP, C-reactive protein; SUA, serum uric acid.

P and r values were from Spearman correlation analyses.

Statistically significant p-values were expressed with bold.

### Association Between PrFV and SUA

**Figure 2:**
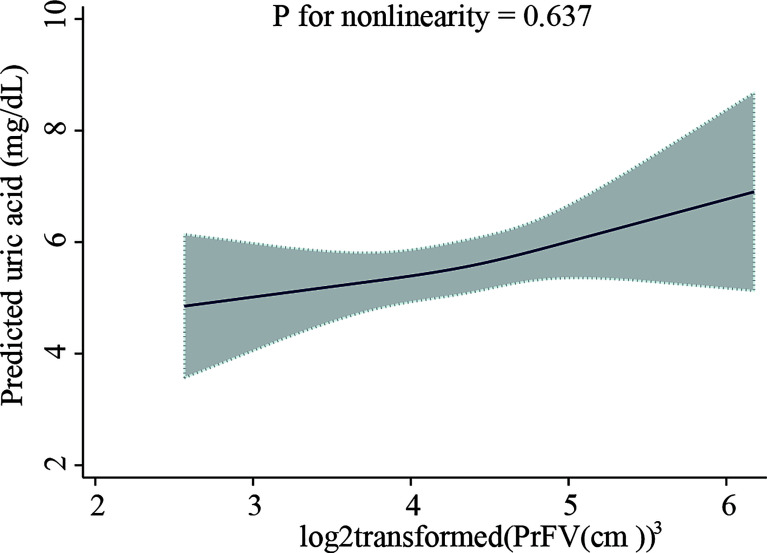
Restricted cubic spline plot of the association between perirenal fat volume with serum uric acid level. Perirenal fat volume was log2-transformed. The analyzes were adjusted for age, sex, waist circumference, serum creatinine, and C-reactive protein. The solid line represents the predicted values of the serum uric acid level, and the gray area represents the 95% confidence interval.

The results of the multivariate analyzes are presented in **Table 3**. In the unadjusted model, subjects in the highest PrFV tertile were associated with a higher level of SUA (β=1.86, 95%CI 1.23–2.48) compared with those in the lowest tertile. After adjustment for covariates, this positive association remained in model 2 (tertile3 versus tertile1: β=1.21, 95%CI 0.58-1.85) and model 3 (tertile3 versus tertile1: β=0.99, 95%CI 0.35-1.63). *P* for trend were <0.001, <0.001 and 0.005, respectively. There was an approximately 0.53 mg/dL increase in SUA per 1-fold increase in PrFV (β=0.53, 95%CI 0.02-1.04, *P* = 0.041) after adjusted for age, sex, WC, SCr, and CRP. The fully adjusted restricted cubic spline showed that PrFV had a linear association with SUA (*P* for non-linearity=0.637, **Figure 2**). Furthermore, the association between PrFV and SUA was independent of the TC, TG, and BMI values (**Supplementary Table 1**).

**Table 3:** The association between PrFV and SUA.

	Model1 β (95% CI)	Model2 β (95% CI)	Model3 β (95% CI)
Tertiles of PrFV, cm^3^			
Tertile1	Ref.	Ref.	Ref.
Tertile2	0.56(-0.07-1.18)	0.16(-0.43-0.76)	0.00(-0.55-0.55)
Tertile3	1.86(1.23-2.48) ***	1.21(0.58-1.85) ***	0.99(0.35-1.63) **
*P* for trend	**<0.001**	**<0.001**	**0.005**
Log2 [PrFV(cm^3^)]	1.30(0.84-1.76) ***	0.78(0.29-1.27) **	0.53(0.02-1.04) *

PrFV was log2-transformed for fitting the generalized linear regression model.

Model 1: Unadjusted.

Model 2: Adjusted for age and sex.

Model 3: Adjusted for age, sex, waist circumference, serum creatinine, and C-reactive protein.

PrFV, perirenal fat volume; SUA, serum uric acid; CI, confidence interval.

***P < 0.001, **P < 0.01 and *P < 0.05.

Statistically significant p-values were expressed with bold.

## Discussion

To our knowledge, this study was the first evaluation to assess the association of ultrasound-derived PrFV with SUA among Chinese adults. And our results demonstrated that perirenal fat accumulation was independently and positively associated with SUA levels.

Several studies have explored the association between perirenal fat and SUA in patients with chronic kidney disease and/or type 2 diabetes, but the results were not well-established. D’Marco et al. found that the thickness of the perirenal fat was significantly correlated with SUA in patients with a GFR<60 mL/min/1.73m^2^, and patients with type 2 diabetes accounted for 28% of this study population (22). Lamacchia and colleagues investigated combined para- and perirenal fat thickness was independently and positively correlated with elevated SUA levels in patients with type 2 diabetes (24). However, the ultrasonographic methodology in this study could not distinguish between perirenal fat and pararenal fat. Besides, in patients with type 2 diabetes, Fang et al. confirmed that perirenal fat thickness was positively related to SUA in a single correlation analysis (25).

Perirenal fat can be measured by CT and ultrasonography. Although CT is a full-scale measurement, ultrasonography is faster, cheaper, and without radiation. In addition, some researchers proved that inter-operator repeatability assessed by the difference between ultrasonography and CT was just 3.2 percent (21). Thus, ultrasonography is more suitable to quantify perirenal fat in large-scale clinical studies. Instead of the single-thickness measured in the above studies, we presented a novel method of measuring the volume of the inferior perirenal fat in three dimensions using ultrasonography. This novel approach provided a more accurate quantification of perirenal fat because the adipose tissue mainly depots at the lower renal pole (17). Naturally, further studies are needed to verify this method.

Compared with previous studies, we ruled out confounding by diabetes mellitus or chronic kidney disease, as glucometabolic disorder and renal dysfunction influence uric acid metabolism (26, 27). Then we performed qualitative and quantitative analyzes of PrFV, and the data were tested with multiple covariates. In our analysis, we found that SUA level was significantly correlated with BMI and WC, which were common indicators of adiposity in clinical practice, consistent with previous studies (28, 29). However, in multivariable generalized linear models, PrFV had a significant positive correlation with SUA, independent of BMI, WC, and other confounders, highlighting the important role of perirenal fat in uric acid metabolism. For the first time, we described the linear association between PrFV and SUA by fully adjusted restricted cubic spline that there was about a 0.53 mg/dL increase in SUA per 1-fold increase in PrFV (β=0.53, 95%CI 0.02-1.04, *P* for nonlinearity=0.637). These findings help better understand the association of PrFV with SUA.

There were several possible mechanisms that may explain the association between PrFV and SUA. First, the imbalance between urinary urate production and excretion contributes to the elevated level of SUA (30). As the subjects in our study had a normal renal function, overproduction might be the main cause of the strong link between perirenal fat and SUA. Uric acid can be produced and secreted by adipose tissue except for the liver and skeletal muscle (31). Excess adipose tissue characterized by hypoxia and active lipid metabolism increased uric acid synthesis and secretion (28, 32). Second, adiponectin (33)has a negative correlation with SUA (33). Increased perirenal fat accumulation can lead to lower adiponectin production (34), thus resulting in the increasing SUA level. Third, inflammation may play a role. Visceral adipose tissue is strongly correlated with inflammatory burden (35). On the other hand, elevated SUA levels are associated with inflammatory conditions (36–38). Therefore, increased inflammation in subjects with the highest PrFV tertile may be responsible of elevated SUA levels. Furthermore, elevated SUA induces fat accumulation through increased intracellular and mitochondrial oxidative stress (39), forming a vicious circle.

There were certain limitations in this study. We cannot explain the causal relationship between SUA and PrFV considering the design of the cross-sectional study. The sample size was limited as this study was a sub-analysis of previously collected data from 2 clinical studies. As a single-center study, the results may not be representative of the other ethnic population. Therefore, further large-scale prospective cohort studies are required to investigate the association. Moreover, we didn’t collect the dietary information which also may influence the adipogenesis and SUA level (40).

## Conclusions

In conclusion, our study demonstrated a linear and positive association between PrFV and SUA, suggesting a possible role of PrFV in the management of SUA. Further studies are necessary to confirm the associations and underlying mechanisms.

## Data Availability Statement

The original contributions presented in the study are included in the article. Further inquiries can be directed to the corresponding authors.

## Ethics Statement

The studies involving human participants were reviewed and approved by the Institutional Review Board of the First Affiliated Hospital of Nanjing Medical University. The patients/participants provided their written informed consent to participate in this study.

## Author Contributions

MJ: Conceptualization, Formal analysis, Writing-Original draft. ML: Resources, Investigation. CL, LJ, QH, and TW: Investigation. JL: Writing–review, and editing. XK: Writing–review and editing, Supervision. All authors contributed to the article and approved the submitted version.

## Conflict of Interest

The authors declare that the research was conducted in the absence of any commercial or financial relationships that could be construed as a potential conflict of interest.

## Publisher’s Note

All claims expressed in this article are solely those of the authors and do not necessarily represent those of their affiliated organizations, or those of the publisher, the editors and the reviewers. Any product that may be evaluated in this article, or claim that may be made by its manufacturer, is not guaranteed or endorsed by the publisher.
