# Supplementary material for: Perirenal Fat Volume Is Positively Associated With Serum Uric Acid Levels in Chinese Adults
Source: Front Endocrinol (Lausanne). 2022 May 6;13:865009. doi: 10.3389/fendo.2022.865009 (PMC9120634; doi:10.3389/fendo.2022.865009)
Supplement: Supplementary file 1 [file Table_1.docx]

Supplemental Table1. Sensitivity analysis for the association between PrFV and SUA

|  | Model4 β (95% CI) | Model5 β (95% CI) | Model6 β (95% CI) |
| --- | --- | --- | --- |
| Tertiles of PrFV, cm^3^ |  |  |  |
| Tertile1 | Ref. | Ref. | Ref. |
| Tertile2 | -0.07(-0.62-0.49) | -0.07(-0.61-0.46) | 0.00(-0.55-0.55) |
| Tertile3 | 1.04(0.40-1.68) ** | 1.07(0.51-1.63) *** | 1.03(0.41-1.65) ** |
| *P* for trend | 0.004 | <0.001 | 0.002 |
| Log2(PrFV(cm^3^)) | 0.54(0.03-1.05) * | 0.61(0.15-1.06) * | 0.59(0.09-1.10) * |

PrFV was log2-transformed for fitting the generalized linear regression model.

PrFV: perirenal fat volume, SUA: serum uric acid, CI: confidence interval.

Model 4: Adjusted for age, sex, serum creatinine, C-reactive protein, total cholesterol, and

and waist circumference.

Model 5: Adjusted for age, sex, serum creatinine, C-reactive protein, total cholesterol, and

triglycerides.

Model 6: Adjusted for age, sex, serum creatinine, C-reactive protein, and body mass index.

****P* < 0.001, ***P* < 0.01 and **P* < 0.05.
